# A Nudge Theory–Based Mobile Health Intervention for Self-Management in Young and Middle-Aged Patients With Hypertension: Mixed Methods Pilot Study

**DOI:** 10.2196/95444

**Published:** 2026-09-10

**Authors:** Yuanyuan Yang, Wei Xu, Yan Zhang, Xue Yang, Linling Li, Jinlong Yang

**Affiliations:** 1 Department of Cardiovascular Medicine, People's Hospital of Leshan, No. 639, Huian Road, Shizhong District, Leshan, Sichuan, 614000, China, 86 08332119336; 2 Department of Nursing, People's Hospital of Leshan, Leshan, Sichuan, China

**Keywords:** hypertension, self-management, mobile health, nudge theory, patient participation

## Abstract

**Background:**

Young and middle-aged patients with hypertension often show poor self-management due to limited cognitive bandwidth. Mobile health apps using behavioral economics may be helpful for these patients.

**Objective:**

This study aimed to evaluate the preliminary effectiveness and usability of a nudge-based mobile app (LeYi) for improving self-management in young and middle-aged patients with hypertension.

**Methods:**

This mixed-methods pilot study used a single-group pre-post design. Thirty participants completed a 24-week intervention. Outcomes were assessed using validated scales and interviews.

**Results:**

Self-management scores showed significant pre-to-post improvements (median change from 68.0 to 108.5; *z*=4.788; *P*<.001), as did eHealth literacy (median change from 15.5 to 34.0; *z*=4.544; *P*<.001). Usability was favorable (median user version of the Mobile App Rating Scale score 4.5, IQR 4.0-5.0). Thematic analysis identified 6 themes that complemented the quantitative findings and highlighted technology adaptation challenges.

**Conclusions:**

The LeYi app may be feasible and acceptable for enhancing self-management in young and middle-aged patients with hypertension. However, a larger randomized controlled trial is needed to determine its efficacy. Embedding behavioral economics into mobile health is a promising approach to support patient engagement.

## Introduction

Cardiovascular disease continues to be the principal cause of mortality globally, with hypertension representing its most significant modifiable risk factor [1]. Of particular concern is the shifting epidemiological profile of hypertension, which demonstrates a marked trend toward younger age groups. In China, the prevalence of hypertension among adults younger than 45 years has increased substantially, rising from 11.3% to 28.8% [2]. This demographic presents distinct challenges within cardiovascular care frameworks. These individuals are frequently characterized by insidious symptom presentation and considerable occupational and lifestyle pressures, and they often exhibit “therapeutic inertia” in managing a chronic condition. Conventional care models, predominantly reliant on periodic outpatient reviews, are often ill-suited to the rhythms of contemporary life, resulting in fragmented delivery of health education, behavioral monitoring, and psychosocial support, a discontinuity that markedly compromises care efficacy. National surveys and clinical consensus indicate that this population achieves significantly lower rates of treatment adherence and blood pressure (BP) control compared with older patients, underscoring the limitations of existing management paradigms [2,3]. Consequently, the development of innovative care models capable of seamless integration into daily routines while providing proactive, continuous, and individualized support has emerged as a pressing priority in cardiovascular care.

Self-management constitutes the fundamental basis for achieving sustained hypertension control. Its central aim is to empower patients by equipping them with the requisite knowledge, practical skills, and confidence for effective disease management [4,5]. Nonetheless, the daily reality of hypertension self-management involves a succession of repetitive health choices characterized by delayed rewards, including consistent medication intake, adherence to a restricted sodium diet, and routine BP monitoring. These requirements are intrinsically at odds with innate cognitive biases that favor immediate gratification and the avoidance of short-term effort [6]. This observed disconnect between intention and actual behavior is a key driver of inadequate treatment adherence, which affects nearly half of all patients, consequently diminishing therapeutic outcomes while increasing the likelihood of complications and associated health care expenditure [7,8]. For young and middle-aged individuals, this behavioral execution challenge is further intensified by constrained cognitive bandwidth—the finite mental capacity available for planning and executing tasks—a limitation frequently imposed by demanding professional and personal schedules.

As a leading digital technology, mobile health (mHealth) apps provide a novel pathway to address the aforementioned challenges. By overcoming spatial and temporal limitations, they function as an extension of nursing care and optimize the delivery of health information and behavioral tracking through highly accessible interactive interfaces [9,10]. Although existing hypertension apps provide basic functions, such as decision support and health education, most lack a solid theoretical foundation in behavioral economics. A systematic evaluation of hypertension management apps in China also noted that the majority of apps demonstrate a notable deficiency in providing personalized, structured interventions based on behavioral change theories [11]. This suggests that many apps fail to address the underlying cause of patients’ “knowledge-action gap,” thereby limiting their effectiveness and sustainability.

The “nudge” theory from behavioral economics provides a powerful framework for the precise design of behavior change strategies [12]. This theory posits that individual decision-making is profoundly influenced by seemingly minor environmental cues (referred to as “choice architecture”), rather than being entirely rational [13]. By consciously optimizing the decision-making environment—for example, using preset “default options” to lower the threshold for action or leveraging “social norms” to stimulate conformity motivation—it is possible to guide people gently toward better decisions without restricting their freedom of choice [14,15]. Research has shown that nudge-based interventions can effectively enhance self-management efficacy and adherence among patients with chronic diseases [14]. Researchers have systematically categorized health-related nudge strategies, providing a concrete methodological blueprint for designing interventions [15]. Applying this theory to mHealth interventions for hypertension means transforming apps from passive tools into proactive “behavioral designers.” By simplifying decision-making processes and providing immediate feedback, these apps can compensate for patients’ limited cognitive bandwidth, helping them adhere to health management more easily amidst their busy lives [10,14]. A recent example of a systematically designed mHealth proof-of-concept study is the work by Balderas-Díaz et al [16], who developed an adaptive mHealth system for a psychoeducational intervention.

Despite its promising potential, research that systematically and user-centrically integrates nudge theory into mHealth interventions for young and middle-aged patients with hypertension remains scarce. Most existing studies either lack a robust theoretical framework or fail to use mixed-methods approaches to explore in depth the underlying mechanisms of intervention effectiveness and real-world user experience [11]. Therefore, this study aimed to undertake an explanatory sequential mixed-methods pilot study to develop and evaluate a nudge theory–based mHealth app named “LeYi.” The specific objectives were to examine the feasibility and acceptability of the app in improving self-management behaviors and eHealth literacy among young and middle-aged patients with hypertension, and to integrate quantitative and qualitative data to gain an in-depth understanding of the potential mechanisms through which the intervention promotes health behavior change, the user acceptability of the intervention, and the challenges encountered in practical application. This will provide an evidence-based foundation for developing theoretically grounded, clinically relevant digital health solutions.

## Methods

### Study Design

This study adopted an explanatory sequential mixed-methods design, which integrates quantitative and qualitative evidence in formative digital health research [17,18]. Quantitative pre-post changes were collected through a 24-week single-group pilot intervention of the LeYi app. Subsequently, postintervention usability questionnaires and semistructured interviews were conducted to supplement and interpret numerical findings. This standard sequential mixed-methods procedure helps enrich the understanding of intervention performance and user experience. The study was reported following the CONSORT-EHEALTH checklist (Checklist 1).

### Feasibility Criteria

Based on methodological guidance for pilot and feasibility studies [19], prespecified feasibility criteria were set as follows: (1) recruitment rate ≥50%; (2) retention rate ≥80%; (3) daily monitoring adherence ≥70% (days with at least one BP reading/168 days); and (4) app usability ≥4.0 on the user version of the Mobile App Rating Scale (uMARS; overall quality score; scale 1‐5). These criteria were used to interpret the feasibility and acceptability of the intervention.

### Development of the LeYi App: Design Based on the Intervention Mapping Framework

The LeYi mHealth app was developed using the Intervention Mapping protocol, a systematic 6-phase framework that emphasizes theory- and evidence-based intervention design [20]. This process ensured that all app features were directly aligned with the identified needs of the target population and grounded in established behavioral science theory.

#### Phase 1: Needs Assessment

A comprehensive needs assessment was conducted through literature review and analysis of current practice, which confirmed suboptimal self-management adherence among young and middle-aged patients with hypertension as the core issue. Key determinants identified included the following: (1) limited cognitive bandwidth due to fast-paced lifestyles, leading to forgetfulness and decision fatigue; (2) lack of immediate feedback and visual cues regarding health status, weakening the perceived connection between behavior and outcomes; and (3) insufficient continuity of support between sporadic clinical visits.

#### Phase 2: Formulation of Change Objectives

Based on the needs assessment, specific and measurable change objectives were formulated at two levels: (1) behavioral objectives and (2) determinant-level objectives. The behavioral objectives were to increase the frequency of (1) daily antihypertensive medication intake, (2) weekly self-monitoring of BP, and (3) regular review of personal health data. The determinant-level objectives were to enhance (1) self-efficacy in managing hypertension, (2) knowledge about the consequences of poor control, and (3) environmental cues that can prompt healthy behaviors.

#### Phase 3: Selection of Theory-Based Methods and Practical Strategies

Nudge theory was selected as the core theoretical framework due to its ability to effectively guide decision-making without restricting freedom of choice [13], making it particularly suitable for addressing the “intention-behavior gap” associated with limited cognitive bandwidth. Systematic reviews have corroborated its effectiveness in improving chronic disease self-management [14]. Specific nudge strategies were mapped onto the established change objectives and translated into practical application features.

#### Phase 4: Program Design and Production

The app architecture and user interface were designed by a multidisciplinary team, including cardiovascular nurses, cardiologists, and software engineers. The design prioritized intuitive navigation, visual clarity, and minimal user burden to accommodate the fast-paced lifestyles of the target users. These elements were integrated into a coherent user journey—from initial onboarding with goal-setting commitments to daily tracking and periodic review—as outlined in Table 1.

Following expert panel consultations and discussions with 2 mHealth technicians conducted from December 27, 2023, to May 1, 2024, the LeYi app was developed on the Android platform using the Vue framework and JavaScript programming language. The design and functionality of the LeYi app are described in detail above.

**Table 1.** Mapping of nudge strategies to app features and corresponding change objectives.

Core behavioral objectives	Behavioral determinants	Selected nudge-based intervention strategies	Specific features in the LeYi app	Proposed mechanism of action (behavior change pathway)
1. Improve daily medication adherence	Memory and reminders Perceived long-term benefits (outcome expectations)	Default option/reminder Commitment device Feedback and reinforcement	Smart medication reminder: Personalized timed reminders Medication adherence tracker and report: Patients log intake, generating weekly/monthly visual reports	Reduce forgetting → Build ritual and commitment via logging → Visual reports provide long-term behavioral feedback → Reinforce regular medication-taking habits
2. Increase regular self-monitoring of blood pressure	Self-efficacy Knowledge and skills Feedback and monitoring	Simplification Feedback and monitoring Self-norming	Minimalist logging interface: Simplified data entry Health dashboard: Automatic generation of blood pressure trend charts and fluctuation analysis Monitoring progress display: Shows personal monitoring frequency and compliance rate	Greatly reduce steps for logging → Lower behavioral burden → Gain sense of control and achievement through trend visualization → Motivate sustained effort by referencing personal progress
3. Promote healthy lifestyle (eg, salt restriction and exercise)	Goal-setting and planning ability Knowledge and skills Social support and norms	Goal setting and decomposition Information architecture Social norms/comparison	Personalized goal setting: Guidance to set specific, measurable goals Contextual knowledge push: Data-driven delivery of relevant articles/videos Community challenges and activities: Initiate online challenges/offline events	Translate intention into concrete plans → Provide knowledge tools for goal achievement → Increase engagement and sense of responsibility through social interaction → Promote practice of healthy behaviors
4. Enhance disease knowledge and patient-provider interaction	Health literacy Communication efficacy and channels	Information architecture and framing effect Simplification	Structured health education library: Themed, multimedia, authoritative health knowledge base Secure messaging module: Supports asynchronous text/image communication with the health care team	Provide accessible, easy-to-understand authoritative information → Improve health literacy and self-management capability → Establish a convenient out-of-hospital support channel → Enhance confidence in management and continuity of care
5. Real-time early warning for enhanced self-management of blood pressure	Default setting and early warning Self-management	Automated monitoring and alerting Timely human intervention (feedback)	Intelligent tiered alert system: Thresholds set according to guidelines; sends tiered alerts when blood pressure is abnormal Clinician-side alert notification and manual response: Alert information is synchronized to the clinician’s workstation, enabling proactive intervention via the messaging module after assessment	Establish automated risk monitoring → Immediately raise patient risk awareness and alert clinicians → Trigger professional, personalized human guidance and support → Strengthen patient safety and appropriate coping ability in urgent situations

#### Phase 5: Planning for Adoption, Implementation, and Sustainability

A detailed implementation protocol was developed for the pilot study. This encompassed standardized usage guidelines for participants, a dedicated troubleshooting manual for research assistants, and a structured technical support plan. The intervention was explicitly designed to complement routine clinical care rather than replace it, thereby ensuring its potential for sustainable future integration.

#### Phase 6: Planning for Evaluation

The evaluation plan was structured following an explanatory sequential mixed-methods design. Quantitative pre-post measures, including the Self-Management Behavior Scale and the eHealth Literacy Scale (eHEALS), were selected to assess preliminary signals of change. Concurrently, qualitative interviews and usability surveys using the uMARS were planned to evaluate implementation processes, user experience, and perceived mechanisms of behavioral change. This integrated approach ensures a comprehensive understanding of the program’s feasibility, acceptability, and overall impact.

### Participants and Setting

This study was conducted from September 1, 2024, to October 22, 2025, in the cardiology outpatient clinic of a tertiary care hospital in Leshan, China.

The inclusion criteria were as follows: (1) age 18‐59 years; (2) diagnosis of essential hypertension according to the Chinese Guidelines for the Prevention and Treatment of Hypertension [21]; (3) current prescription of at least one antihypertensive medication; (4) owning a smartphone and proficiency in basic operations (eg, installing apps and receiving messages); and (5) voluntary participation and providing written informed consent.

The exclusion criteria were as follows: (1) presence of secondary hypertension; (2) history of severe psychiatric illness or cognitive impairment that would preclude study participation; (3) history of drug or alcohol abuse; (4) plan to become pregnant, current pregnancy, or breastfeeding during the study period; (5) concurrent participation in other clinical trials that could interfere with this study; and (6) any other conditions deemed unsuitable for participation by the researchers (eg, life expectancy of less than 1 year).

A convenience sampling method was used, and a total of 30 eligible patients were enrolled. According to sample size guidelines for pilot studies, a sample of 12 to 30 participants per group is considered appropriate for pilot studies primarily focusing on feasibility assessment and effect size estimation [22]. Moreover, this study adopted an explanatory sequential mixed-methods design, with in-depth interviews planned for all participants. A sample size of 30 patients also provides a robust foundation for achieving thematic saturation in qualitative data analysis. All enrolled participants completed the 24-week intervention and subsequent assessments, with no dropouts, yielding a final analytical sample of 30 participants.

### Intervention Implementation

All participants were instructed to install the LeYi app on their smartphones. Prior to the intervention, trained researchers provided standardized, face-to-face, one-on-one instructions (approximately 15‐20 min), focusing on the operation of core functions. The 24-week intervention was delivered entirely through the app, which was developed following the Intervention Mapping framework and systematically integrated nudge strategies, including default options, immediate feedback, and social norms, to guide health behaviors by optimizing the digital decision-making environment.

The intervention’s core mechanism involved decomposing self-management goals into daily micro-tasks with low cognitive load and reinforcing positive behavior through real-time feedback and visualization. According to the Chinese Guidelines for the Management of Hypertension (2024 Revision), the recommended home BP monitoring target is systolic BP (SBP) <135 mmHg and diastolic BP (DBP) <85 mmHg. In this study, BP control was defined accordingly. Participants were guided to use 5 core functional modules, each designed based on the aforementioned theoretical principles (see Table 1 for strategy mapping and detailed feature descriptions). Screenshots of the app interface are provided in Multimedia Appendix 1. The key interaction logic operated as follows: establishing medication routines through standardized adherence support; enabling visual tracking of health data via structured BP monitoring; translating health goals into concrete actions through contextualized lifestyle guidance; facilitating early risk identification and professional intervention through a tiered alert and clinician response mechanism; and providing ongoing information and support through closed-loop patient-provider communication and education. Together, these 5 modules constituted a continuous management cycle encompassing monitoring, alerting, intervention, and support.

To ensure smooth initiation of the intervention, the research team provided proactive technical adaptation support via telephone or in-app messages during the first 2 weeks, addressing initial barriers such as login difficulties and operational challenges. Throughout the intervention period, the app operated autonomously according to preset logic (eg, medication reminders and report generation). The critical early warning workflow functioned as follows: when a user-entered BP value reached the tiered alert threshold defined in accordance with the Chinese Guidelines for the Prevention and Treatment of Hypertension, the system issued an alert to the user and simultaneously transmitted the warning information to the responsible clinician’s terminal. The clinician then conducted a manual assessment and delivered personalized guidance via the secure in-app messaging module. The research team reviewed anonymized, aggregated BP reading availability on a weekly basis to monitor overall engagement at a macro level.

The app was designed to function as a “digital bridge” connecting patients’ daily self-management with professional medical support. By integrating intelligent assistance with human collaboration, it aimed to enhance the feasibility, safety, and sustainability of self-management.

### App Workflow and Clinician Response

Patients submitted home BP readings through the app 4 times daily (before meals and at bedtime). The system automatically graded BP levels using color-coded labels: red for BP ≥140/90 mmHg and orange for BP 130-139/80‐89 mmHg. When an alert was triggered, the clinical team reviewed the patient’s data, assessed the reading, checked for potential measurement errors, and delivered individualized guidance within 24 hours. For readings suspected to be affected by improper measurement technique, patients received retraining on standard measurement methods and were asked to remeasure. Risk grades were reassessed based on the remeasurement results. For confirmed abnormal readings, clinicians provided advice through the secure in-app messaging module. In cases of severe hypertension or symptoms, medical staff contacted the patient immediately by telephone and recommended nearby medical attention. All patients underwent BP reassessment after the intervention. Patients with controlled BP were placed under long-term follow-up, while those with recurrent red alerts were managed as high-risk cases and arranged for offline outpatient visits.

### Measures

#### Participant Baseline Sociodemographic and Clinical Characteristics

Participant sociodemographic characteristics, including age, gender, marital status, and educational level, were collected using a self-administered questionnaire. Clinical characteristics collected included smoking status, alcohol consumption status, and the number of antihypertensive medications prescribed.

#### Outcome Variables

To evaluate the preliminary effectiveness of the LeYi app, we assessed patients’ self-management behaviors and eHealth literacy before and after the intervention.

Self-management behaviors were measured using the Self-Management Behavior Scale for Young and Middle-Aged Patients with Hypertension, developed by Gong et al [23] specifically for the Chinese population. This scale comprises 25 items across 4 dimensions, each rated on a 5-point Likert scale, with total scores ranging from 25 to 125 [23]. Higher scores indicate better self-management. The scale demonstrated high internal consistency in the original study (Cronbach α=.930) [23] and acceptable reliability in our study (Cronbach α=.792).

The eHEALS, originally developed by Norman and Skinner [24], was used to assess participants’ ability to understand, evaluate, and use eHealth information and digital health tools. The Chinese version of the scale, adapted and validated by Guo et al [25], consists of 8 items across 3 dimensions, each rated on a 5-point Likert scale ranging from 1 (“strongly disagree”) to 5 (“strongly agree”). Higher total scores indicate greater eHealth literacy (possible range: 8‐40) [25]. The Chinese version of eHEALS has demonstrated good reliability in previous studies, with a Cronbach α value of .885 [25], and showed internal consistency in our study (Cronbach α=.965).

#### Usability Testing

After the intervention, the usability of the LeYi app was evaluated by integrating quantitative and qualitative data collected from individual interviews. The quality of the mHealth app was assessed using the uMARS. This scale comprises 16 items across 4 subscales (engagement, functionality, esthetics, and information), together with an additional 10 items under 2 further subscales (subjective app quality and perceived impact). Each item is rated on a 5-point scale (range 1-5), with higher scores indicating higher app quality. Items on subjective app quality and perceived impact are reported separately.

To obtain qualitative data on usability, a researcher conducted face-to-face semistructured interviews using an interview guide and audio recordings. The interview guide was developed through iterative discussions within the research team, informed by the study objectives and a review of relevant literature. The questions were designed to elicit participants’ actual experiences with the app during the intervention and addressed four core areas: (1) overall impression of the app; (2) satisfaction with its usability; (3) perceived usability shortcomings; and (4) changes in self-care behaviors after using the app.

During the interviews, a trained research assistant took field notes to summarize the content. Participants’ confidentiality and anonymity were strictly protected throughout the process. All interviews were transcribed verbatim and cross-checked for accuracy. Participants were identified by unique codes in the transcripts, and any personal identifying information was excluded from the analysis. Any discrepancies in data processing were resolved through team discussion until consensus was reached.

### Study Procedure

Two research assistants were responsible for baseline data collection. They assisted participants in operating their smartphones and successfully installing the LeYi app. Pretest questionnaires were completed in a quiet, private room within the cardiology outpatient clinic.

After the 24-week intervention, the same research assistants collected posttest questionnaire data at the same venue. Subsequently, one-on-one semistructured interviews were conducted with each participant by a cardiovascular nurse who had received training in qualitative research methods. No relationship was established with participants prior to study commencement; participants were informed only of the researcher’s professional role and the study purpose. Only the researcher and participant were present during the interviews.

The interview guide was developed based on a literature review and was pilot tested with 2 patients (not included in the final sample) to refine the questions. Each interview lasted approximately 30‐45 minutes and was audio-recorded. Field notes were taken during and after each interview. All interviews were transcribed verbatim, and transcripts were returned to participants for member checking to verify accuracy. Data collection continued until thematic saturation was achieved, meaning no new themes emerged from subsequent interviews. Interviews focused on participants’ experiences with the app and their perceived changes in self-management behaviors.

### Ethical Considerations

This study was approved by the Institutional Review Board of the authors’ affiliated institution (approval number: LYLL[2024]KY107). All participants provided written informed consent prior to enrolment. Participants were assured that their identity and all personal information would be kept strictly confidential. To ensure confidentiality, unique identification numbers were assigned to each participant and used throughout the data analysis process in place of their names.

### Data Analysis

Statistical analyses were performed using IBM SPSS Statistics for Windows, version 27.0 (IBM Corp). All participant data were entered and systematically verified for consistency. Categorical variables are presented as frequencies and percentages. Continuous variables, which were not normally distributed as determined by the Kolmogorov-Smirnov test, are reported as medians with IQRs. Within-group comparisons between pre- and postintervention were conducted using the nonparametric Wilcoxon signed-rank test. Statistical significance was set at a 2-tailed *P* value of <.05.

Although the primary inferential analysis used nonparametric Wilcoxon signed-rank tests due to nonnormal distributions, means, 95% CIs, and Cohen *d* values are also reported as descriptive effect size measures to facilitate comparison with other studies. These estimates are presented as supplementary descriptive statistics and should not be interpreted as parametric inferential results.

For qualitative data, thematic analysis was used to systematically examine the interview transcripts. Two researchers independently performed the analysis; both had substantial knowledge of the patient population through clinical and research experience, as well as expertise in qualitative methodology. Coding and categorization were facilitated using Microsoft Word and Excel. An inductive and iterative approach was adopted. The researchers familiarized themselves with the data by repeatedly reading the transcripts, after which initial codes were generated. These codes were subsequently collated into potential themes [26]. Themes were then reviewed, defined, and named following verification of coding accuracy and data completeness. Throughout the process, the 2 researchers worked independently and held regular discussions to compare coding structures, refine thematic classifications, and reconcile interpretations until consensus was achieved.

Quantitative and qualitative data pertaining to the usability of the LeYi app were analyzed separately within the mixed-methods framework. An integrated interpretation of these findings will be presented in the Discussion section.

## Results

### Feasibility Outcomes

All prespecified feasibility criteria were met or approached. The recruitment rate was 75% (30/40), exceeding the 50% criterion. Retention was 100% (30/30), exceeding the 80% criterion. Daily monitoring adherence was 67.5%, approaching the 70% criterion. App usability was favorable, with a median uMARS overall quality score of 4.5 (IQR 4.3‐4.7), exceeding the 4.0 criterion. These findings suggest that the intervention was feasible and acceptable in this pilot context.

### Participant Baseline Sociodemographic and Clinical Characteristics

Of the 40 individuals invited to participate, 30 (75%) provided written informed consent and were enrolled. No intervention-related adverse events were reported during the study period.

Table 2 shows the baseline characteristics of the 30 enrolled participants. The mean age was 39.17 (SD 7.89; range 27‐58) years, with the largest subgroup aged 31‐40 years (17/30, 57%). Of the 30 participants, 19 (63%) were male and 11 (37%) were female. The majority were married (27/30, 90%). Regarding educational attainment, over half of the participants (17/30, 57%) held a bachelor’s degree or higher, whereas 27% (8/30) had completed high school or vocational secondary education or below. Current smokers comprised 33% (10/30) of the sample, nonsmokers comprised 60% (18/30), and former smokers comprised 7% (2/30). Moreover, 53% (16/30) reported current alcohol consumption. With respect to antihypertensive regimens, half of the patients (15/30, 50%) were prescribed a single agent, 14 (47%) were taking 2 or 3 agents, and only 1 (3%) was prescribed 4 or more agents.

**Table 2.** Baseline sociodemographic and clinical characteristics of the patients (N=30).

Characteristic	Value
Age (years)	
Mean (SD)	39.17 (7.89)
Range	27‐58
Age group (years), n (%)	
≤30	3 (10)
31‐40	17 (57)
41‐50	7 (23)
51‐59	3 (10)
Gender, n (%)	
Male	19 (63)
Female	11 (37）
Marital status, n (%)	
Single	3 (10)
Married	27 (90)
Education level, n (%)	
Middle school or below	7 (23)
High school/technical secondary school	1 (3)
Junior college	5 (17)
Bachelor’s degree	17 (57)
Smoking status, n (%)	
Yes	10 (33)
No	18 (60)
Previous smoker	2 (7)
Alcohol drinking, n (%)	
No	14 (47)
Yes	16 (53)
Number of medications, n (%)	
1	15 (50)
2‐3	14 (47)
≥4	1 (3)

### Intervention Effects on Self-Management Behavior and eHealth Literacy

**Figure 1.**
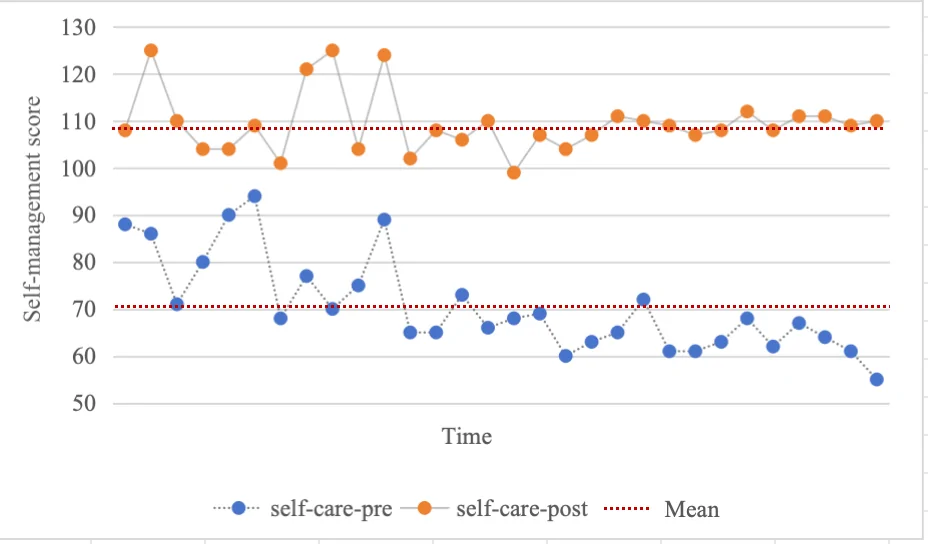
Individual changes in self-management behavior (N=30).

**Figure 2.**
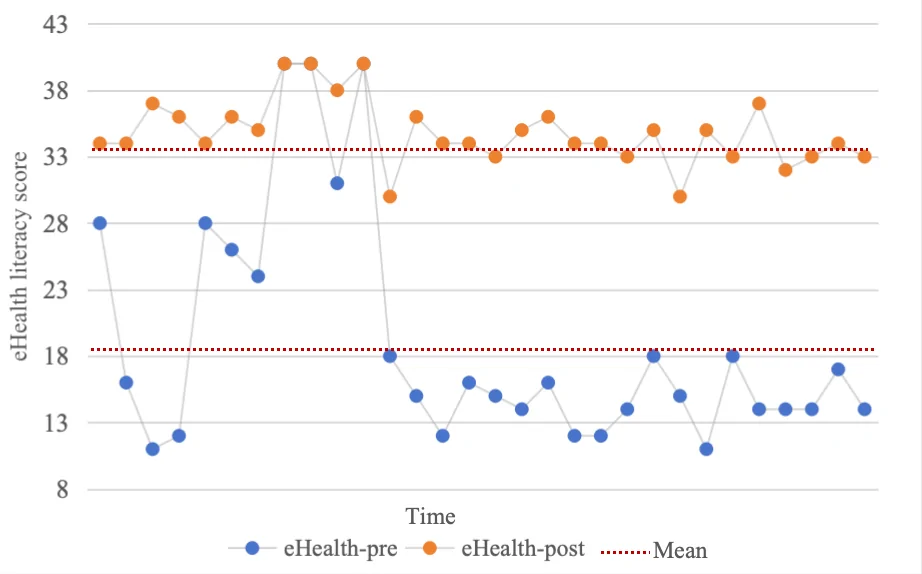
Individual changes in eHealth literacy (N=30).

As shown in Table 3, self-management behavior showed a significant pre-to-post improvement. The median score increased from 68.0 (IQR 63.0‐75.5) at baseline to 108.5 (IQR 105.5‐111.0) postintervention. The mean change was 38.93 (95% CI 35.05-42.81), with a large effect size (Cohen *d*=3.75; *z*=4.788; *P*<.001). Similarly, eHealth literacy showed a significant improvement. The median score increased from 15.5 (IQR 14.0‐24.5) to 34.0 (IQR 33.0‐36.0), with a mean change of 15.67 (95% CI 12.88-18.46) and a large effect size (Cohen *d*=2.10; *z*=4.544; *P*<.001). However, due to the uncontrolled single-group design, these improvements cannot be attributed specifically to the intervention. Graphs for self-management behavior and eHealth literacy are provided in Figures 1 and 2, respectively.

**Table 3.** Comparison of pre- and posttest results in patients with hypertension who received the 24-week intervention (N=30).

Outcome variable	Pretest, median (IQR)	Posttest, median (IQR)	Mean change, value (95% CI)	Cohen *d*	*z* ^a^	*P* value
Self-Management Behavior Scale score	68.0 (63.0‐75.5)	108.5 (105.5‐111.0)	38.93 (35.05-42.81)	3.75	4.788	<.001
eHealth Literacy Scale score	15.5 (14.0‐24.5)	34.0 (33.0‐36.0)	15.67 (12.88-18.46)	2.10	4.544	<.001

a Wilcoxon signed-rank test.

### Usability of the LeYi App

#### Quantitative Usability Results

Table 4 presents the quantitative usability results assessed using the uMARS. The LeYi app achieved a favorable overall quality score, with a median of 4.5 (IQR 4.3‐4.7). Among the 4 subscales (engagement, functionality, esthetics, and information), the information subscale received the highest median rating (4.3, IQR 4.0‐4.5). The engagement subscale scored relatively lower (median 3.2, IQR 3.0‐3.5).

Regarding subjective app quality, participants expressed a strong willingness to recommend the app (median 4.0), while their willingness to pay was more conservative (median 3.0). In the perceived impact domain, items, such as “awareness” (median 4.0) and “behavior change” (median 4.0), were rated highly, suggesting the app’s potential in enhancing health-related cognition and promoting behavioral change.

During the study period, the primary usability barrier reported by some participants was difficulty logging in due to forgotten passwords. No other significant issues affecting app usability were identified.

In summary, the LeYi app was well received by users in terms of information quality, visual design, and core functionality. Although there is room for improvement in user engagement and personalization, the app shows good overall user experience and has potential for supporting health management.

**Table 4.** Scores of the user version of the Mobile Application Rating Scale (uMARS) after the 24-week intervention (N=30).

Item	Score, median (IQR)
App quality (total)	4.5 (4.3‐4.7)
Engagement (total)	3.2 (3.0‐3.5)
Entertainment	3.0 (3.0‐4.0)
Interest	3.0 (3.0‐4.0)
Customization	2.0 (2.0‐3.0)
Interactivity	3.0 (3.0‐4.0)
Target group	4.0 (4.0‐5.0)
Functionality (total)	4.0 (3.7‐4.1)
Performance	4.0 (3.0‐4.0)
Ease of use	4.0 (4.0‐5.0)
Navigation	4.0 (3.8‐4.0)
Gestural design	4.0 (3.0‐4.0)
Esthetic (total)	4.0 (3.7‐4.3)
Layout	4.0 (3.0‐4.0)
Graphics	4.0 (4.0‐5.0)
Visual appeal	4.0 (4.0‐5.0)
Information (total)	4.3 (4.0‐4.5)
Quality of information	5.0 (4.0‐5.0)
Quantity of information	4.0 (4.0‐5.0)
Visual information	4.0 (4.0‐4.3)
Credibility	4.0 (4.0‐5.0)
Subjective app quality	3.5 (3.3‐3.8)
Recommend?	4.0 (3.8‐5.0)
Estimated frequency	3.5 (3.0‐4.0)
Would you pay?	3.0 (3.0‐3.3)
Overall rating	3.0 (3.0‐4.0)
Perceived impact	3.9 (3.8‐4.2)
Awareness	4.0 (4.0‐5.0)
Knowledge	4.0 (3.0‐4.0)
Attitudes	4.0 (3.0‐4.0)
Intention to change	4.0 (3.0‐4.0)
Help seeking	4.0 (3.8‐4.0)
Behavior change	4.0 (4.0‐5.0)

#### BP Monitoring Frequency and Control

Based on available app records, we further analyzed BP monitoring frequency and BP control rates among the 30 participants over the 24-week intervention period (168 days). BP control was defined according to the Chinese Guidelines for the Management of Hypertension (2024 Revision), with the target for home BP monitoring set at SBP <135 mmHg and DBP <85 mmHg.

A total of 7356 BP readings were recorded across all participants, with a mean of 245.2 readings per participant (range 112‐420). The mean number of days with at least one BP recording was 113.4 (range 80‐165) days. The mean daily monitoring adherence, calculated as the number of days with at least one BP reading divided by the total intervention days (168 days), was 67.5% (range 47.6%‐98.2%).

The mean SBP was 127.8 (range 117‐136) mmHg, and the mean DBP was 73.2 (range 60‐87) mmHg. The mean BP control rate, defined as the proportion of readings meeting the target of SBP <135 mmHg and DBP <85 mmHg, was 41.6% (range 20.4%‐66.7%). A total of 3112 out of 7356 readings (42.3%) achieved BP control.

Due to technical limitations in backend data extraction, login frequency and core function usage data were not systematically captured. Thus, we focused our analysis on BP monitoring frequency and control rates, which were reliably derived from available app records.

#### Qualitative Usability Results

Six primary themes were identified during the individual interviews, as outlined in Multimedia Appendix 2. These themes are presented below.

##### Theme 1: Visualized Health Monitoring Enhances Self-Awareness and Facilitates Behavioral Internalization

Most participants reported that features, such as BP trend charts, medication adherence logs, and health education progress tracking, markedly improved their awareness and comprehension of their own health status. The data visualization mechanism not only reinforced patients’ intuitive understanding of disease progression but also enabled them to actively link abstract BP readings with concrete daily behaviors, such as diet and physical activity, thereby fostering the internalization and routinization of healthy practices.

*Recording my blood pressure every day and observing the waveform changes helped me better understand how my lifestyle affects my blood pressure. It also encouraged me to consciously adjust my diet and exercise habits.*

[Participant #21]

##### Theme 2: Goal-Oriented Interactive Design Strengthens Motivation for Behavioral Maintenance

Features, including continuous check-ins, task completion feedback, personalized goal setting, and BP anomaly alerts, formed a positive reinforcement loop. By achieving short-term, quantifiable goals (eg, daily monitoring), patients experienced a heightened sense of self-efficacy and accomplishment, which in turn reinforced their intrinsic motivation and sustained engagement in long-term self-management.

*Every time I completed a measurement and saw a stable blood pressure trend chart, I felt a real sense of achievement. That positive feedback made me more motivated to keep using the app for self-management.*

[Participant #6]

##### Theme 3: Continuous Patient-Provider Communication Optimizes Disease Management Support

The in-app communication model with attending physicians enhanced the continuity of clinical interactions. Participants reported that this reduced the need for nonurgent hospital visits while enabling more personalized and coherent guidance tailored to their ongoing condition.

*Being able to communicate directly with my attending physician—who already knows my history—through the app saved me from frequent trips to the hospital and gave me more targeted and consistent advice.*

[Participant #18]

##### Theme 4: Information Support and Real-Time Feedback Improve Psychological Adaptation and Health Literacy

The integration of continuous monitoring, automated alerts, and targeted knowledge dissemination created a multilayered information support system. This strengthened participants’ confidence in managing their condition and alleviated anxiety associated with disease uncertainty.

*Seeing my blood pressure trends in real time and getting prompt feedback from my doctor made me feel much more confident about managing my condition [...].*

[Participant #13]

##### Theme 5: Technological Adaptability and Perceived Behavioral Burden Constrain Long-Term Adherence

Some participants—particularly older adults with lower digital literacy—encountered initial difficulties in adapting to the technology. In addition, the requirement for daily data entry was perceived by certain users as a behavioral burden, potentially undermining sustained engagement.

*Having to measure and record my blood pressure every day sometimes felt like an extra chore. On busy days, it could be quite stressful.*

[Participant #2]

*For those of us who aren’t very comfortable with smartphones, even though we recognize the app’s value, sticking with it over the long term remains a challenge.*

[Participant #11]

##### Theme 6: Desired Enhancements in Functionality and Interactivity Point to Future Optimization Directions

Participants articulated clear expectations for future iterations of the app, including automatic data synchronization, more sophisticated personalized feedback, integration of social features, and diversification of patient-provider communication modalities.

*I hope the app can automatically sync data from my smart bracelet to reduce the hassle of manual entry, and also offer more personalized suggestions based on my health data.*

[Participant #8]

*It would be great if future versions could support voice or video calls with doctors—that would make consultations much more direct and efficient.*

[Participant #24]

Table 5 presents a joint display linking key quantitative findings to the 6 qualitative themes derived from participant interviews.

**Table 5.** Integration of quantitative and qualitative results.

Quantitative finding	Qualitative theme	Representative quotation	Concordance
Self-management behavior scores significantly increased from baseline (median 68.0, IQR 63.0‐75.5) to postintervention (median 108.5, IQR 105.5‐111.0; *z*=4.788; *P*<.001).	Theme 1: Visualized health monitoring enhances self-awareness and facilitates behavioral internalization	“Recording my blood pressure every day and observing the waveform changes helped me better understand how my lifestyle affects my blood pressure. It also encouraged me to consciously adjust my diet and exercise habits.” (Participant #21)	Concordant
eHealth literacy scores significantly increased from baseline (median 15.5, IQR 14.0‐24.5) to postintervention (median 34.0, IQR 33.0‐36.0; *z*=4.544; *P*<.001).	Theme 4: Information support and real-time feedback improve psychological adaptation and health literacy	“Seeing my blood pressure trends in real time and getting prompt feedback from my doctor made me feel much more confident about managing my condition.” (Participant #13)	Concordant
High overall app acceptability (uMARS^a^ total median 4.5, IQR 4.3‐4.7); the Information subscale achieved the highest score (median 4.3).	Theme 3: Continuous patient-provider communication optimizes disease management support	“Being able to communicate directly with my attending physician—who already knows my history—through the app saved me from frequent trips to the hospital and gave me more targeted and consistent advice.” (Participant #18)	Concordant
Mean daily monitoring adherence of 67.5% (range 47.6%‐98.2%); higher adherence was linked to stronger user motivation. Wide variation in the blood pressure control rate (20.4%‐66.7%), with lower adherence associated with poor blood pressure control.	Theme 2: Goal-oriented interactive design strengthens motivation for behavioral maintenance Theme 5: Technological adaptability and perceived behavioral burden constrain long-term adherence	“Every time I completed a measurement and saw a stable blood pressure trend chart, I felt a real sense of achievement. That positive feedback made me more motivated to keep using the app for self-management.” (Participant #6) “Having to measure and record my blood pressure every day sometimes felt like an extra chore. On busy days, it could be quite stressful.” (Participant #2)	Partially discordant
Users put forward multiple demands for function upgrades, including automatic data synchronization and personalized feedback.	Theme 6: Desired enhancements in functionality and interactivity point to future optimization directions	“I hope the app can automatically sync data from my smart bracelet to reduce the hassle of manual entry, and also offer more personalized suggestions based on my health data.” (Participant #8)	Concordant

a uMARS: user version of the Mobile App Rating Scale.

## Discussion

### Principal Findings

This pilot study, using an explanatory sequential mixed-methods design, suggests that the nudge theory–based LeYi app exhibits good feasibility and shows preliminary trends in self-management among young and middle-aged patients with hypertension. Quantitative findings revealed notable changes after the intervention: after 24 weeks, patients’ self-management behaviors (*z*=4.788; *P*<.001) and eHealth literacy (*z*=4.544; *P*<.001) showed significant pre-to-post increases relative to baseline. Moreover, the app received a favorable usability rating, with a median uMARS overall quality score of 4.5, indicating that its high information quality and ease of use provided a foundational basis for the observed changes. Consistent with previous research examining self-management in elderly patients with hypertension [27], our study extends these findings to a younger population and explores the potential of theory-driven mHealth interventions to support self-management behaviors.

The joint display (Table 5) illustrates how the qualitative themes consistently supported and contextualized the quantitative improvements. Participants who reported greater self-management improvement also described how visualized health monitoring (theme 1) helped them internalize healthy behaviors, while those with higher eHealth literacy gains frequently mentioned the value of real-time feedback and information support (theme 4). These findings were concordant and mutually reinforcing. However, a partially discordant case also emerged. Despite the overall improvements in self-management and eHealth literacy, some participants with lower daily monitoring adherence reported that daily data entry felt burdensome (theme 5). This suggests that while the app was beneficial for many, the requirement for manual entry may pose a barrier to long-term engagement for a subset of users. This discordance highlights the need for future iterations to reduce user burden, for example, by integrating automated data synchronization.

Importantly, the qualitative findings elucidated the mechanisms behind these quantitative results. Thematic analysis of the interview data revealed 6 key dimensions related to intervention performance: at the cognitive level, visualized monitoring facilitated internalization of health awareness; at the behavioral and motivational levels, goal-oriented design strengthened motivation for sustained engagement; and at the support and contextual levels, continuous patient-provider interaction and information feedback optimized the support system, while technological burden emerged as a notable challenge. These insights collectively point toward future optimization directions, including the demand for expanded functionality. The qualitative evidence thus complements and corroborates the quantitative results, together constructing a comprehensive evidence chain that illustrates how this theory-driven mHealth intervention supports self-management through multidimensional pathways.

The core findings of this study provide empirical support for the application of nudge theory in chronic disease self-management, particularly within digital health interventions targeting young and middle-aged populations. The notable changes in patients’ self-management behaviors directly corroborate a central tenet of nudge theory: that optimizing the “choice architecture,” rather than imposing mandates, can effectively guide individuals characterized by “bounded rationality” toward healthier decisions [13]. More importantly, this study demonstrated how these abstract nudge principles can be operationalized into a coherent set of actionable digital intervention strategies, specifically tailored to address the behavioral challenges prevalent among young and middle-aged patients with hypertension, such as limited cognitive bandwidth and a propensity for immediate gratification.

The design of the LeYi app systematically integrated multiple categories of health-related nudge tools, as categorized by Blumenthal-Barby and Burroughs [15]. Commitment devices were embedded through goal setting and daily check-ins; environmental priming and immediate feedback were facilitated via data visualization and alerts; and decision costs were minimized by simplifying the recording process through default options. Qualitative insights from user interviews provided direct evidence regarding these strategies’ roles in overcoming common behavioral inertia and execution difficulties. Participants frequently reported that “reminders helped me build a habit” and that “visualizing the data made me clearer about what to do.” These findings resonate with the broader body of systematic evidence regarding nudge strategies and health-related behaviors, including guideline adherence among health care professionals [28], thereby underscoring the generalizability of these principles across varied contexts and actors.

The findings of this study offer a promising approach to address the specific self-management challenges encountered by young and middle-aged patients with hypertension. Due to their fast-paced lifestyles and pronounced health perception biases, sustaining engagement and ensuring long-term adherence in this population have remained persistent challenges in the field of mHealth [29]. The observed trends in this study show that an intervention grounded in nudge theory can help address these challenges [13,14]. Its value stems from the translation of behavioral economic principles into actionable digital strategies that systematically reshape patients’ decision-making environments.

First, by simplifying decision-making processes—through features such as rapid data entry—the cognitive and operational costs of initiating behavior are reduced, thereby accommodating individuals’ fragmented schedules and limited cognitive bandwidth [10,13]. Second, by providing immediate and intuitive feedback (eg, trend charts), the intervention transforms long-term, often imperceptible health benefits into short-term, visible signals, thereby counteracting inherent perception biases [30]. Finally, the integration of gentle, external reminders as preconfigured environmental cues actively bridges the intention-behavior gap [13,15]. The core of this multicomponent strategy is neither coercive nor didactic; rather, it creates a digital environment in which healthier choices become more convenient, salient, and accessible, thereby gently guiding users to overcome bounded rationality and behavioral inertia.

The high ratings for “functionality” and “ease of use,” together with users’ qualitative feedback emphasizing the app’s “simplicity of operation,” are not incidental findings. Rather, they represent direct outcomes of the design philosophy outlined above, collectively confirming that a design ethos centered on “reducing burden and enhancing efficiency” aligns closely with the pragmatic needs of this population, who prioritize utility and practicality [10]. Notably, the relatively modest scores for “engagement” (eg, entertainment value) did not affect overall performance. On the contrary, this observation suggests that for the young and middle-aged patients with hypertension targeted in this study, a tool that is efficient, reliable, and seamlessly integrable into daily routines is valued more highly than one featuring elaborate entertainment functions [11,29]. This insight advances our understanding of design priorities for digital health interventions tailored to this specific demographic.

This study provides preliminary evidence regarding the potential of a nudge theory–based mHealth intervention for supporting self-management among young and middle-aged patients with hypertension. By reshaping the decision-making environment to bridge the “knowledge-attitude-practice” gap, this approach demonstrates clear advantages in terms of accessibility, acceptability, and potential sustainability. Consequently, it offers both a valuable theoretical foundation and a viable digital tool prototype for designing public health interventions aiming to help improve hypertension control rates in this population [31].

Specifically, this model could be explored as a complementary component within existing hypertension prevention and control frameworks. In response to the limited reach of traditional in-person services among young and middle-aged adults, this intervention attempts to supplement these services through digital means. Its daily monitoring functionalities may contribute to earlier identification of health risks, while structured reminders and support could potentially facilitate adherence during the initial phases of treatment [29]. Furthermore, continuous data tracking and light-touch interactions may offer additional support for some patients during intervals between conventional outpatient visits. Naturally, these potential benefits warrant further validation in broader populations and over extended follow-up periods [31].

Moreover, this mHealth intervention may have potential for scalability. However, its cost-effectiveness remains theoretical, as no economic data were collected in this pilot study, and formal health economic assessments are needed in future research. mHealth interventions typically entail low marginal costs following initial development, providing an economic rationale for large-scale implementation [32]. Given the serious consequences of poorly controlled hypertension and its substantial socioeconomic burden, the potential benefits of preventive digital interventions merit careful consideration [33]. It should be noted that, as an initial pilot exploration, the primary value of this study lies in establishing the feasibility of the intervention and generating preliminary evidence to inform future larger-scale research, including rigorous health economic evaluations [34].

In summary, the thoughtful integration of such behaviorally informed digital tools into comprehensive hypertension management strategies—subject to further research and evaluation—may offer insights into addressing certain gaps in current service delivery. This approach also contributes to accumulating evidence and experience for developing innovative chronic disease prevention and control strategies suited to the digital era.

### Clinical Implications for Cardiovascular Nursing

The findings of this pilot study offer several practical implications for cardiovascular nurses working with young and middle-aged patients with hypertension.

First, the LeYi app can serve as a tool that extends nursing care beyond clinical settings. Nurses can introduce the app during discharge education, guiding patients to use medication reminders and BP monitoring features. During follow-up telephone consultations, nurses can review the app’s data dashboard with patients to track adherence and identify areas needing additional support.

Second, the visualized health data generated by the app, such as BP trend charts and medication adherence logs, can facilitate nurse-patient shared decision-making. By reviewing these visualizations together, nurses can help patients better understand the relationship between their daily behaviors (eg, diet and physical activity) and BP control, thereby reinforcing motivation for sustained self-management.

Third, the tiered alert system integrated with clinician notification demonstrates how nurses can leverage technology for proactive intervention. When patients receive automated alerts for abnormal BP readings, nurses can follow up promptly via the app’s secure messaging module, providing timely guidance and preventing complications.

However, the technological adaptability challenges reported by some participants highlight an important nursing role: providing initial guidance and ongoing technical support. Nurses should assess patients’ digital literacy at baseline, offer personalized instructions on app use, and remain available to address operational difficulties, particularly for those less comfortable with smartphone technology.

By incorporating such theory-driven digital tools into routine practice, nurses can play a pivotal role in bridging the intention-behavior gap and promoting sustainable self-management in this challenging population.

### Strengths and Limitations

A principal strength of this study lies in the development of the LeYi app, a locally tailored mHealth intervention for young and middle-aged patients with hypertension. Designed to fit their lifestyle and usage needs, the app uses the local language, which improves cultural adaptability and user acceptability.

This study also has several limitations. First, it adopted a single-group pre-post design with no control group. Observed changes are only preliminary within-participant trends and cannot be attributed entirely to the intervention. The Hawthorne effect, regression to the mean, and concurrent routine care may also have contributed to the observed changes. Second, this was a single-center pilot study with a small sample size (n=30), limiting the generalizability of the findings. Large-sample multicenter randomized controlled trials are needed for further validation. Third, outcomes mainly depended on self-reported scales, and the 24-week follow-up was too short to assess long-term behavioral changes and clinical endpoints. More objective indicators and longer follow-ups are required in future work. Fourth, participants needed basic smartphone skills, which may have caused inequity due to differences in digital access and literacy.

Despite the above limitations, the findings of this pilot study indicate that theory-driven mHealth tools can support hypertension self-management. Moreover, the study provides practical experience for shifting hypertension care toward proactive digital prevention.

### Conclusions

The findings of this pilot study suggest that the LeYi app, a mobile health intervention grounded in nudge theory, may be feasible and acceptable for supporting self-management behaviors and eHealth literacy among young and middle-aged patients with hypertension. With features, including simplified decision-making, real-time feedback, and strengthened patient-provider interaction, the app applies behavioral economic principles to build a user-friendly digital choice architecture. This design helps users cope with behavioral inertia and cognitive biases in self-management and offers a noncoercive way to guide health behaviors. Given the single-group design without a control arm, these findings are only preliminary trends. Larger randomized controlled trials are required to further verify the intervention’s actual efficacy.

This study provides preliminary evidence for adopting theory-driven digital health tools as a complementary approach in hypertension management. Such tools may help optimize hypertension control. Further studies with larger samples and longer follow-ups are needed to explore the generalizability and long-term performance of this intervention. Overall, this work offers useful theoretical references and practical implications for developing user-oriented, localized mHealth tools for hypertension and promotes digital development in chronic disease prevention and management.

## Supplementary material

10.2196/95444 Multimedia Appendix 1 Sample screenshots from the LeYi app.

10.2196/95444 Multimedia Appendix 2 Thematic analysis of qualitative data.

10.2196/95444 Checklist 1 CONSORT‐EHEALTH checklist (V 1.6.1).

## Abbreviations

BP: blood pressure
DBP: diastolic blood pressure
eHEALS: eHealth Literacy Scale
mHealth: mobile health
SBP: systolic blood pressure
uMARS: user version of the Mobile App Rating Scale
