# Supplementary material for: A Nudge Theory–Based Mobile Health Intervention for Self-Management in Young and Middle-Aged Patients With Hypertension: Mixed Methods Pilot Study
Source: JMIR Form Res. 2026 Sep 10;10:e95444. doi: 10.2196/95444 (PMC13561441; doi:10.2196/95444)
Supplement: Supplementary file 1 [file formative-v10-e95444-s001.pdf]

Supplementary Figure S1. Sample Screenshots From the LeYi App.

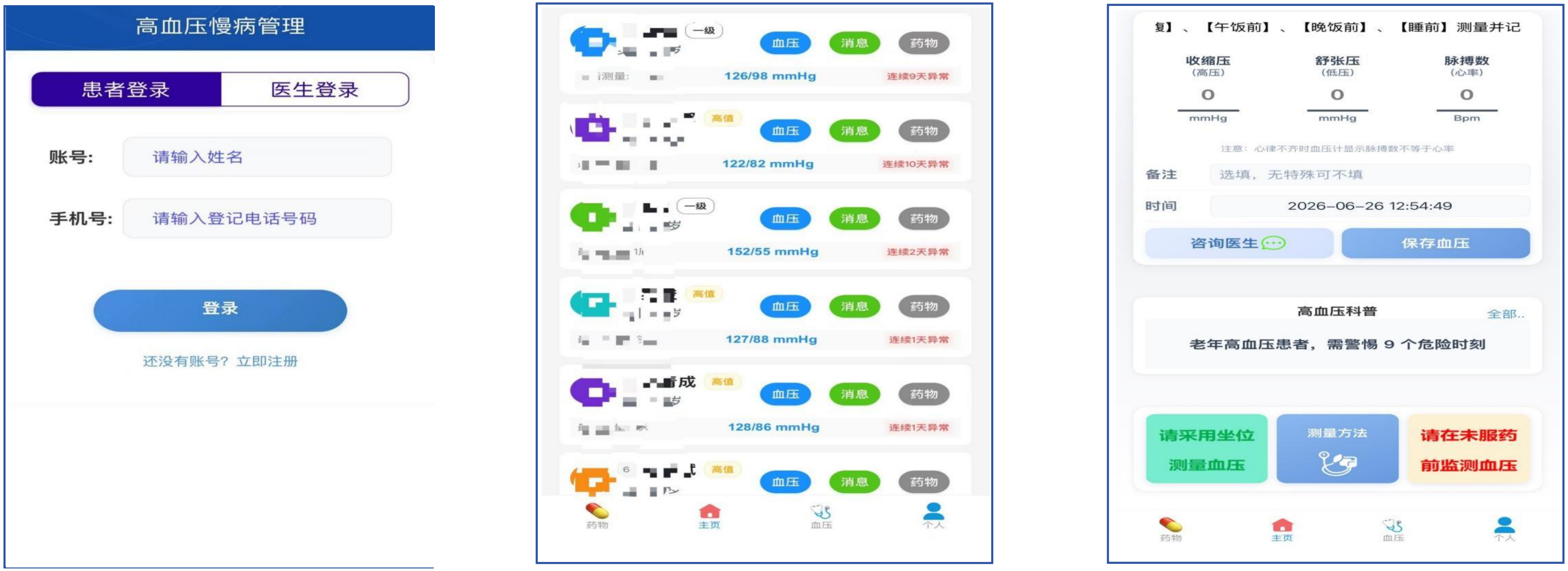

A B C

Note. (A) Login page with patient and clinician access; (B) Alert message list showing abnormal BP readings (C) Blood pressure entry interface;

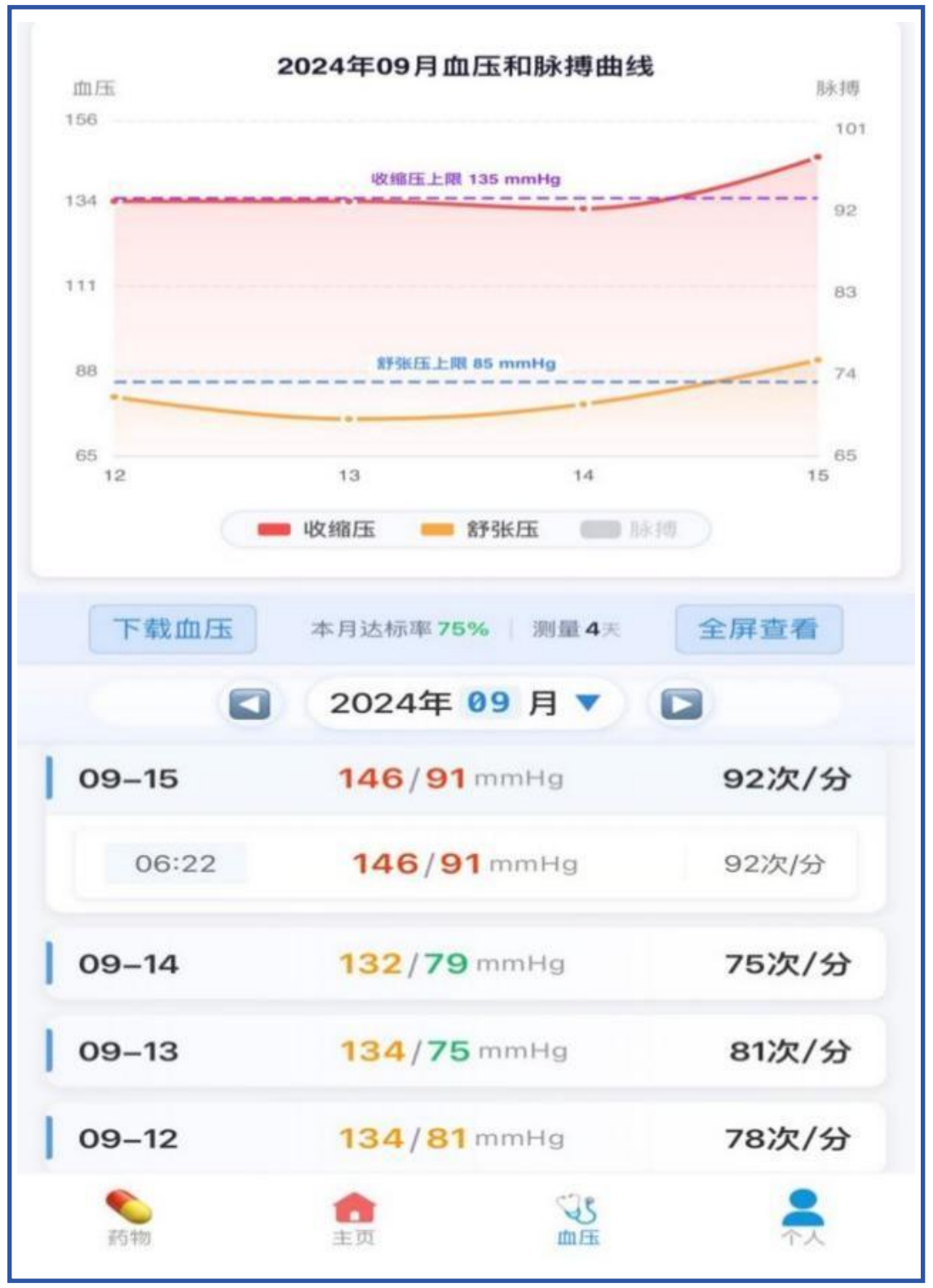

D

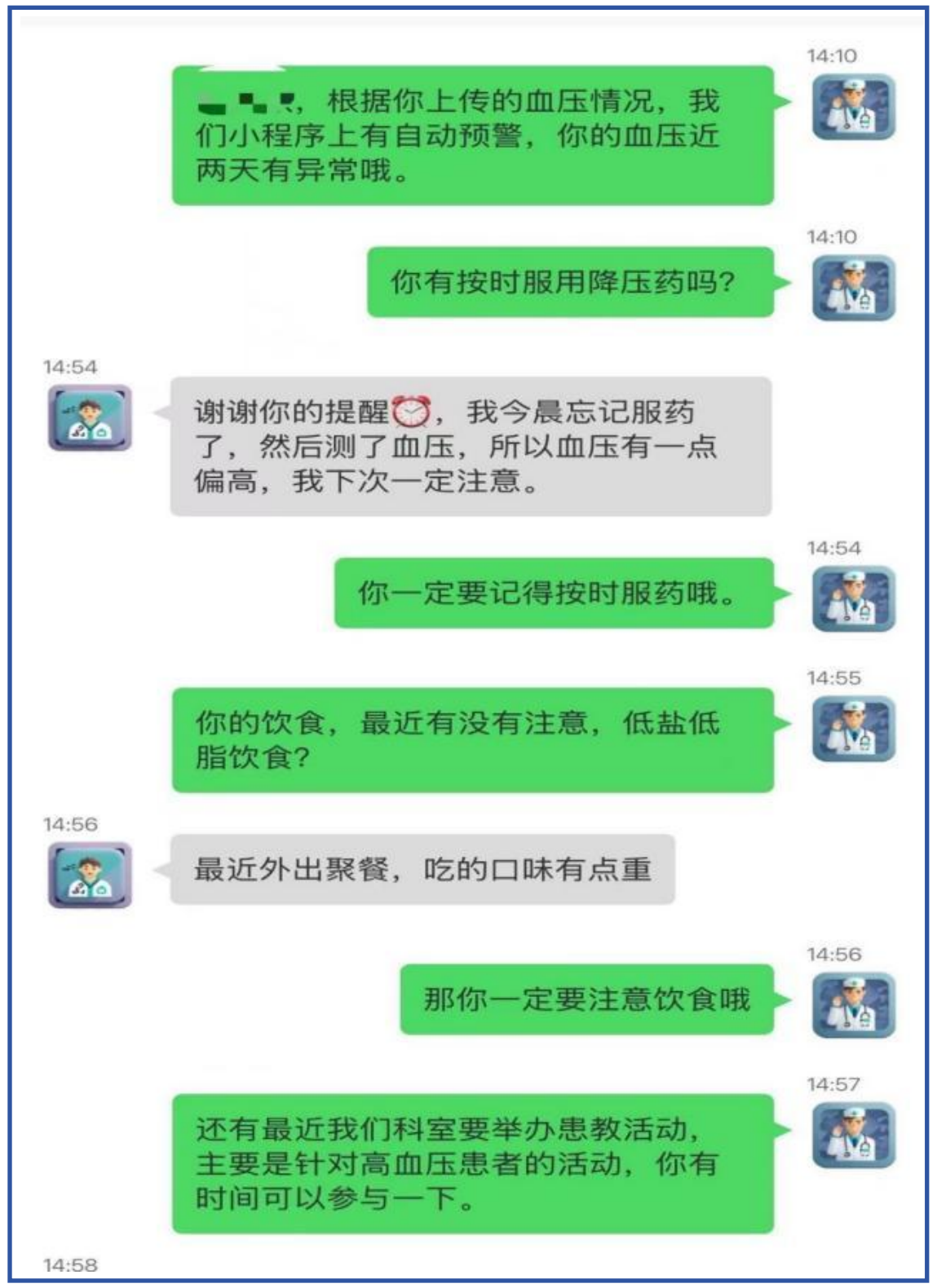

E

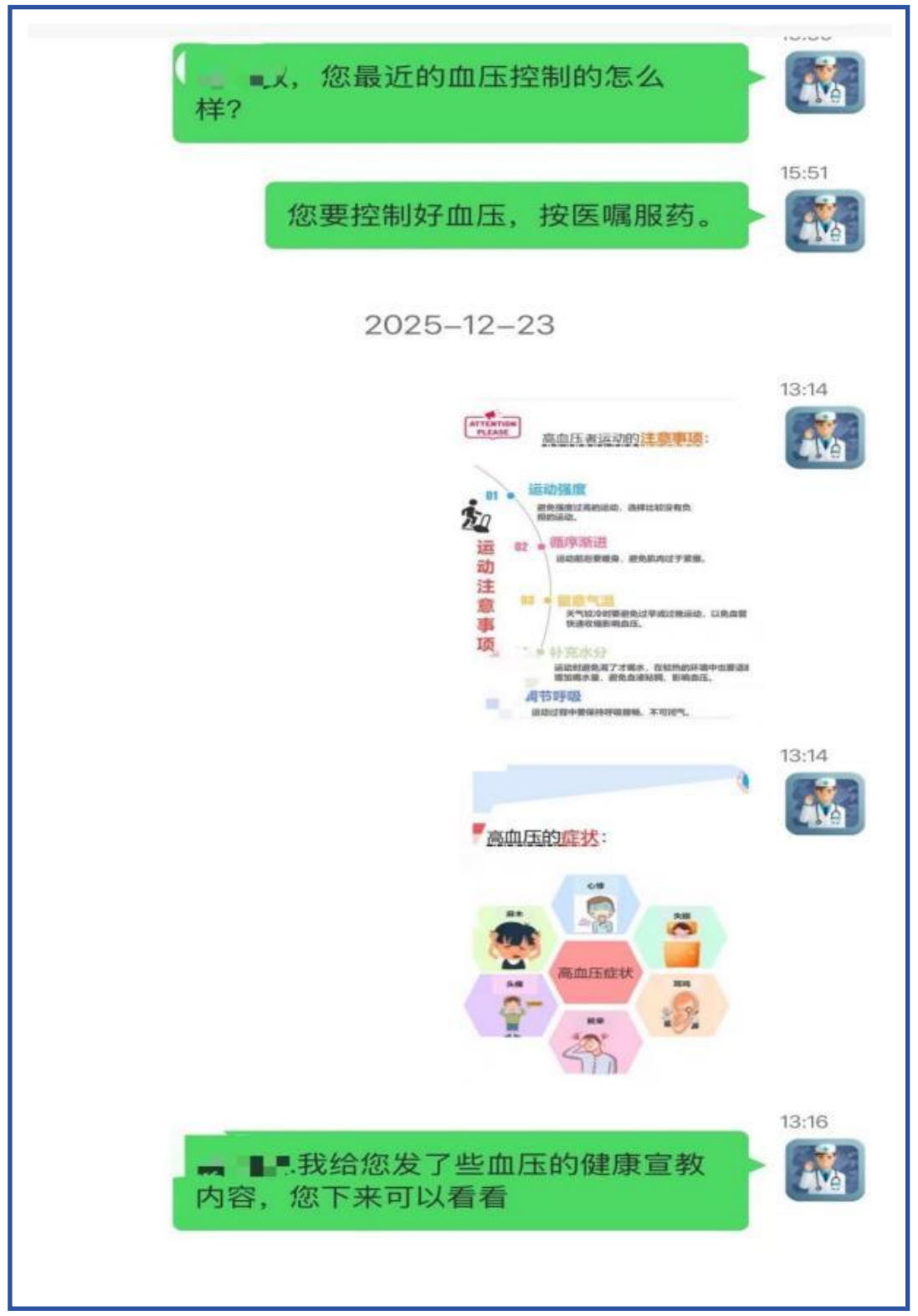

F

Note. (D) Blood pressure trend chart; (E) Secure messaging module; (F) Clinician intervention interface.
