# Supplementary material for: A Nudge Theory–Based Mobile Health Intervention for Self-Management in Young and Middle-Aged Patients With Hypertension: Mixed Methods Pilot Study
Source: JMIR Form Res. 2026 Sep 10;10:e95444. doi: 10.2196/95444 (PMC13561441; doi:10.2196/95444)
Supplement: Supplementary file 2 [file formative-v10-e95444-s002.docx]

****Supplementary Table S1 – Thematic Analysis of Qualitative Data****

| Theme | Sub-theme | Category |
| --- | --- | --- |
| Visualised Health Monitoring Enhances Self-Awareness and Facilitates Behavioral Internalisation | Data visualization heightens awareness of health status | - Observing health changes intuitively through blood pressure trend charts - Tracking medication adherence via digital logging - Reinforcing knowledge acquisition through health education progress tracking |
|  | Data-driven insights promote internalisation of healthy behaviours | - Actively linking abstract blood pressure values with concrete behaviors such as diet and exercise - Consciously adjusting lifestyle habits based on data feedback - Gradually transforming health management behaviors into daily routines |
| Goal-Oriented Interactive Design Strengthens Motivation for Behavioral Maintenance | Interactive features provide immediate positive reinforcement | - Continuous check-ins demonstrate Behavioral consistency - Task completion feedback offers instant recognition - Blood pressure anomaly alerts enable timely intervention |
|  | Goal achievement reinforces intrinsic motivation | - Accomplishing short-term, quantifiable goals (e.g., daily monitoring) - Gaining self-efficacy from goal attainment - Experiencing a sense of achievement from positive feedback (e.g., viewing stable trend charts) - Strengthening willingness to engage in long-term self-management |
| Continuous Patient–Provider Communication Optimizes Disease Management Support | Online communication improves healthcare accessibility | - Direct communication with attending physicians via the app - Reducing the need for hospital visits for non-urgent matters |
|  | Continuous interaction enhances quality of guidance | - Receiving personalized advice based on longitudinal medical history - Obtaining coherent and consistent professional guidance |
| Information Support and Real‑Time Feedback Improve Psychological Adaptation and Health Literacy | Systematic information support enhances sense of control | - Continuous monitoring provides a comprehensive view of health status - Alert functions signal potential risks - Regular knowledge dissemination improves disease awareness |
|  | Timely feedback alleviates anxiety and boosts confidence | - Viewing health data trends in real time - Receiving timely professional interpretation and feedback - Enhancing confidence in one's ability to manage the condition - Alleviating anxiety associated with disease uncertainty |
| Technological Adaptability and Perceived Behavioral Burden Constrain Long‑Term Adherence | Digital literacy disparities create usage barriers | - Some older users face initial operational difficulties - Unfamiliarity with smartphone application functions - Long-term adherence remains challenging despite recognizing the app's value |
|  | Daily management tasks impose subjective burden | - Requiring daily measurements and recordings - Perceived as an additional task during busy periods - Task-related pressure may affect long-term engagement |
| Desired Enhancements in Functionality and Interactivity Point to Future Optimization Directions | Demand for automation and intelligent features | - Expectation of automatic data synchronization with wearable devices - Expectation of more personalized feedback based on individual health data |
|  | Demand for diversified and interactive communication modes | - Expectation of introducing social features or peer support - Expectation of richer communication modalities with healthcare providers, such as voice or video calls |
