# Supplementary material for: A Nudge Theory–Based Mobile Health Intervention for Self-Management in Young and Middle-Aged Patients With Hypertension: Mixed Methods Pilot Study
Source: JMIR Form Res. 2026 Sep 10;10:e95444. doi: 10.2196/95444 (PMC13561441; doi:10.2196/95444)
Supplement: Supplementary file 3 [file formative-v10-e95444-s003.docx]

**CONSORT‐EHEALTH checklist (V.1.6.1)**

| **Section/Topic** | **Item No.** | **CONSORT* Checklist Item** | **EHEALTH Extensions** | **Reported on page** |
| --- | --- | --- | --- | --- |
| **Title and abstract** | 1a | Title and abstract | i) Identify the mode of delivery in the title | 1 |
|  | 1a |  | ii) Mention non-web-based components in title | N/A |
|  | 1a |  | iii) Mention primary condition/target group in title | 1 |
|  | 1b | Structured summary of trial design, methods, results, and conclusions | i) Key features/functionalities/components of the intervention and comparator in abstract | **1** |
|  | 1b |  | ii) Level of human involvement in abstract | 7 |
|  | 1b |  | iii) Open vs. closed recruitment, web-based vs. face-to-face assessments | 6 |
|  | 1b |  | iv) Results in abstract must contain use data | 1 |
|  | 1b |  | v) Conclusions for negative trials | N/A |
| **Introduction** | 2a | Scientific background and explanation of rationale | i) Describe problem and type of system/solution | 2-4 |
|  | 2a |  | ii) Scientific background, rationale | 2-4 |
| **Methods** | 3a | Description of trial design | No EHEALTH-specific additions | 4 |
|  | 3b | Important changes to methods after trial commencement | i) Bug fixes, downtimes, content changes | N/A |
|  | 4a | Eligibility criteria for participants | i) Computer/Internet literacy eligibility | 6 |
|  | 4a |  | ii) Open vs. closed, web-based vs. face-to-face assessments | 6 |
|  | 4a |  | iii) Information given during recruitment | 10 |
|  | 4a |  | iv) Outcomes self-assessed online | 7-8 |
|  | 4a |  | v) Institutional affiliations displayed | N/A |
|  | 4a |  | vi) Developers/sponsors affiliations | 27 |
|  | 5 | Interventions for each group with sufficient details to allow replication | i) History/development process and formative evaluations | 4-5 |
|  | 5 |  | ii) Revisions and updating | N/A |
|  | 5 |  | iii) Quality assurance methods | 5 |
|  | 5 |  | iv) Replicability (source code, screenshots, flowcharts) | 4-6, Table 1 (6) |
|  | 5 |  | v) Digital preservation (URL, archive) | N/A |
|  | 5 |  | vi) Access (how participants accessed, payment, membership) | 6 |
|  | 5 |  | vii) Mode of delivery, features/functionalities, theoretical framework | 4-6, Table 1 (6) |
|  | 5 |  | viii) Use parameters (intended doses, timing) | 7 |
|  | 5 |  | ix) Level of human involvement | 7 |
|  | 5 |  | x) Prompts/reminders used | 7 |
|  | 5 |  | xi) Co-interventions | N/A |
|  | 6a | Completely defined pre-specified primary and secondary outcome measures | i) Online questionnaire validation | 7-8 |
|  | 6a |  | ii) Use/adoption metrics (logins, logfile analysis) | 7 |
|  | 6a |  | iii) Qualitative feedback | 8 |
|  | 6b | Any changes to trial outcomes after commencement | No EHEALTH-specific additions | N/A |
|  | 7a | How sample size was determined | i) Expected attrition in sample size calculation | 6 |
|  | 7b | Interim analyses and stopping guidelines | No EHEALTH-specific additions | N/A |
|  | 8a-10 | Randomization, allocation, implementation | No EHEALTH-specific additions | N/A |
|  | 11a | Blinding | i) Specify who was blinded | 10 |
|  | 11a |  | ii) Informed consent procedures creating biases | 10 |
|  | 11b | Similarity of interventions | No EHEALTH-specific additions | N/A |
|  | 12a | Statistical methods | i) Imputation techniques for attrition/missing values | N/A |
|  | 12a |  | No EHEALTH-specific additions | 11 |
|  | 12b | Additional analyses | No EHEALTH-specific additions | N/A |
| **Ethics & Informed Consent** | X26 | (Not a CONSORT item) | i) Ethics committee approval | 10 |
|  | X26 |  | ii) Informed consent procedures | 10 |
|  | X26 |  | iii) Safety and security procedures, privacy considerations | 10 |
| **Results** | 13a | Participant flow (diagram strongly recommended) | i) Attrition diagram (use over time) | N/A |
|  | 13b | Losses and exclusions after randomization | i) Secular events | N/A |
|  | 14a | Dates defining periods of recruitment and follow-up | i) Digital divide demographics | 12-13, Table 2 (12) |
|  | 14b | Why trial ended | No EHEALTH-specific additions | 12 |
|  | 15 | Baseline demographic and clinical characteristics | i) Multiple denominators and definitions of use | 12-13, Table 2 (12) |
|  | 16 | Numbers analyzed | i) Primary intent-to-treat | 12 |
|  | 16 |  | ii) Secondary analyses comparing only users | N/A |
|  | 16 |  | iii) Presentation of process outcomes | 7 |
|  | 17a | For each group, outcomes and effect sizes | i) Presentation of process outcomes | 13-14, Table 3 (13), Table 4 (14) |
|  | 17b | Absolute and relative effect sizes | No EHEALTH-specific additions | 13-14 |
|  | 18 | Other analyses performed | i) Subgroup analysis of users only | N/A |
|  | 19 | All important harms or unintended effects | i) Privacy breaches, technical problems | 12 |
|  | 19 |  | ii) Qualitative feedback on strengths/shortcomings | 15-17 |
| **Discussion** | 20 | Trial limitations | i) Typical limitations in ehealth trials | 22-23 |
|  | 21 | Generalizability | i) Generalizability to other populations | 22-23 |
|  | 21 |  | ii) Differences in routine application setting | 22-23 |
|  | 22 | Interpretation | i) Restate questions and summarize answers | 17 |
|  | 22 |  | ii) Unanswered questions, future research | 23 |
| **Other Information** | 23 | Registration | No EHEALTH-specific additions | N/A |
|  | 24 | Protocol | No EHEALTH-specific additions | N/A |
|  | 25 | Funding | No EHEALTH-specific additions | 27 |
|  |  | X27 | (Not a CONSORT item) | i) Relation of study team to system evaluated |

* CONSORT = Consolidated Standards of Reporting Trials [10]

** NPT = non pharmacological treatment (CONSORT extension) [11]

References

1. Baker TB, Gustafson DH, Shaw B, Hawkins R, Pingree S, Roberts L, Strecher V. Relevance of CONSORT reporting criteria for research on eHealth interventions. Patient Educ Couns.

2010 Dec;81 Suppl:S77-86

2. Talmon J, Ammenwerth E, Brender J, de Keizer N, Nykänen P, Rigby M. STARE-HI--Statement on reporting of evaluation studies in Health Informatics. Int J Med Inform. 2009 Jan;78(1):1-9. Epub 2008 Oct 18.

3. Eysenbach G. Issues in evaluating health websites in an Internet-based randomized controlled trial. J Med Internet Res 2002;4(3):e17

4. Blankers M, Koeter MWJ, Schippers GM. Missing Data Approaches in eHealth Research: Simulation Study and a Tutorial for Nonmathematically Inclined Researchers. J Med Internet Res 2010;12(5):e54

5. Eysenbach G. The law of attrition. J Med Internet Res 2005;7(1):e11

6. Proudfoot et al. Establishing Guidelines for Executing and Reporting Internet Intervention Research. Cognitive Behaviour Therapy (forthcoming)

7. Webb TL, Joseph J, Yardley L, Michie S. Using the Internet to Promote Health Behavior Change: A Systematic Review and Meta-analysis of the Impact of Theoretical Basis, Use of Behavior Change Techniques, and Mode of Delivery on Efficacy. J Med Internet Res 2010;12(1):e4

8. Cugelman B, Thelwall M, Dawes P. Online Interventions for Social Marketing Health Behavior Change Campaigns: A Meta-Analysis of Psychological Architectures and Adherence Factors. J Med Internet Res 2011;13(1):e17

9. Eysenbach G. Improving the Quality of Web Surveys: The Checklist for Reporting Results of Internet E-Surveys (CHERRIES). J Med Internet Res 2004;6(3):e34

10. Schulz KF, Altman DG, Moher D, for the CONSORT Group (2010) CONSORT 2010 Statement: Updated Guidelines for Reporting Parallel Group Randomised Trials. PLoS Med 7(3): e1000251

11. Boutron I, Moher D, Altman DG, Schulz K, Ravaud P, for the CONSORT group. Extending the CONSORT Statement to randomized trials of nonpharmacologic treatment:

explanation and elaboration. Ann Intern Med. 2008:295-309
